# An indicator of the view of time passing: the development and validation of the metacognitive knowledge of time passing scale (MKTPS) in Chinese college students

**DOI:** 10.3389/fpsyg.2024.1364166

**Published:** 2024-08-16

**Authors:** Xide Yu, Cheng Lu, Yaju Ma, Li Huang, Chenyang Wu

**Affiliations:** ^1^Department of Applied Psychology, School of Education Science, Guangdong Polytechnic Normal University, Guangzhou, China; ^2^Student Affairs Office, Nanfang College, Guangzhou, Guangdong, China; ^3^College of Education Science, Weinan Normal University, Shaanxi, China; ^4^Student Counselor Work Center, School of Education Science, Guangdong Polytechnic Normal University, Guangzhou, China

**Keywords:** metacognition, passage of time, view of lifetime passing, metacognitive knowledge of time passing scale, reliability, validity

## Abstract

How we view the passage of past time determines how we face time itself as well as our futures, which has a strong impact particularly during the highly creative and malleable college years. Chinese culture cherishes time deeply, and for centuries there has been a tradition of “educating children and youth to inspect the passage of time.” However, in today’s age of information and intelligence, time has shown a trend toward fragmentation. How do contemporary Chinese college students view the passage of time, and what structures or content does it contain? The answer to this question remains uncertain, necessitating further exploration. Following Flavell’s theory of metacognitive knowledge (MK), we adopted a semi-structured interview method and used the results to first outline the basic structure of Chinese college students’ view of time passing, identifying four major aspects: priming aftereffect, life touching, positive promotion, and negative inhibition. Then, using the initial four-dimensional structure as a starting point, we developed the Metacognitive Knowledge of Time Passing Scale (MKTPS), and carried out exploratory factor analysis and confirmatory factor analysis to test its fit. The results showed that the four-factor scale and its 22 items had a good fit to the data. Third, the reliability and validity of the self-developed scale were tested. The results show that the internal consistency, split-half, and retest reliability of the MKTPS are good (all *r*s > 0.60). The construct validity of the MKTPS is also good (*r*_between subscales_ is 0.33–0.60, *r*_between subscales and total scale_ is 0.64–0.87), the convergent validity with Zimbardo’s negative past time perspective is high (*r* = 0.37), and the discriminant validity with Zimbardo’s future time perspective is significant (*r* = 0.18). Regarding criterion correlation validity, the total scores of the MKTPS have a significantly higher positive correlation with those of the time management disposition (TMD) scale (*r* = 0.45). Future points for studying the view of time passing in adults of all ages and across cultures field and shortcomings of the current study are also discussed.

## Introduction

For each day we as human live, we are 1 day closer to death. From this perspective, time is a non-renewable and irreversible property – once it is lost, it can never be regained. Unlike tangible material wealth, however, time is invisible, yet our minds must make sense of its passage and management. Therefore, for human individuals, the view of time passing is a necessary skill in managing our wealth of time, which is crucial to living our lives fully. From a societal perspective, a people or nation’s concept of the passage of time will shape its unique time culture (Flaherty et al., 2005; Flaherty, 2018). In Western societies where timekeeping technology is popular, the passage of time is linearly forward and irreversible. Therefore, in perceiving time, these cultures place more emphasis on “present” and “future” than on “past.” Research in developmental psychology has revealed that Western children typically acquire an understanding of the “present” around the age of 2, while their comprehension of the intricate relationship between the “present,” “past,” and “future” may not fully develop until they reach the age of 5 or even later (Tillman et al., 2017). Conversely, Chinese children tend to grasp these temporal connections at approximately 4 years old (Yu et al., 2019). Additionally, it is worth mentioning that most Western children are more inclined to perceive time as a linear progression from left to right (Tillman et al., 2018). However, in Eastern countries that stress events, quality, and periodicity, time and its passage are cyclic. In Eastern cultures, time has a broader concept of “present” or “nowness,” while also regarding both “past” and “future” as equally important (Li-Jun et al., 2023). This may be one of the reasons that Chinese culture attaches great importance to the passing of time, that is, Chinese people are likely to look forward to a better future by reflecting upon time passing.

Passage of time as a theme is not new within the schools of physics or philosophy (Fiocco, 2007; Deng, 2013), however in psychology, compared to the duration judgment (DJ), particularly in comparison to the “short duration judgment” encompassing milliseconds, seconds, or even minutes (Droit-Volet, 2013; Droit-Volet et al., 2017; Chan and Saqib, 2022), there remains a relative scarcity of theories and empirical studies on “passage of time judgment (PoTJ).” Therefore, it can be asserted that this field is still in its nascent stage. Overall, existing psychological research on passage of time has focused primarily on four aspects. First, studies have surveyed people’s judgments about the speed of time passing in the past and in the present. For example, researchers used an ecologically valid experience sampling method (ESM) to measure how quickly time was perceived to be passing. The ESM necessitates daily participation from subjects over a specified research period (typically lasting around a week), wherein researcher/s pose relevant questions. Each day, participants are provided with invitations to provide their responses. In two separate ESM studies focusing on the feeling of passage of time, alerts were sent to participants’ smartphones eight times a day for five consecutive days, and participants were asked to complete a short questionnaire each time about the speed of time passing, and their emotional state, arousal level, and attention level (Droit-Volet and Wearden, 2016; Droit-Volet, 2019). The results showed that, in contrast to “common sense” beliefs, no significant difference was evident between younger or older participants in their judgments of how fast time was passing, at least not in terms of their judgments of the current speed of its passing.

The second general aspect current studies on the passage of time have explored is what might influence one’s judgment of the speed of time passing, noting the impact of four specific factors. (1) Emotional state. Generally, individuals perceive time to pass more quickly during positive emotional states and slower during negative emotional states. For example, sadness caused both young and old participants to perceive time as passing more slowly (Droit-Volet and Wearden, 2015, 2016). (2) Attention. The more attention one devote to “time,” the slower it appears to pass, while conversely, it seems to pass more swiftly. Prior studies showed that there is a significant negative correlation between the degree to which one’s attention is captured by present activities and one’s judgment of the current speed of the passage of time, but this applies only to young participants, not to the old. In other words, for young individuals, the more difficult or engaging an activity is, the more their attention is drawn to the activity, and the faster time seems to pass; however, the easier an activity, the less attention one pays to the activity, allowing one to focus more attention on time itself and thus time seems to pass slower (Droit-Volet and Wearden, 2015, 2016). It is worth noting that this underlying principle bears a striking resemblance to the “attentional model” of short duration estimation (prospective timing), wherein excessive allocation of attention resources toward processing “non-temporal information” leads to neglect of “temporal information,” resulting in shortened time estimation (Block and Zakay, 1997; Zakay and Block, 2004; Block et al., 2010). (3) Time pressure. The existence of a substantial positive correlation between time pressure and feeling of the passage of time is apparent. On the one hand, a cross-sectional comparison of past and present time pressures showed that participants often systematically underestimate past time pressures (Janssen et al., 2013). On the other hand, participants who said they were under a great deal of current time pressure reported that time went by quickly in shorter intervals, while those who reported that they had been under great time pressure 10 years previously, but were no longer under that same pressure, reported that the past 10 years had gone by quickly (Janssen et al., 2013). (4) Time perspective. Gallagher (2000) posited that time perspective (TP) serves as the embodiment of the narrative self. The term “narrative self” refers to an individual’s diverse representations of oneself, encompassing the various narratives we construct about our past and future, which involve memories of the past and prospects for the future. Moreover, researchers in psychology who investigate time consciousness also contend that there exists a close association between the “narrative self,” “time perspective,” and the “feeling of the passage of time.” Essentially, feeling of time passing entails an evaluation of how one perceives their present self in relation to their past and future selves. Therefore, it can be considered as a manifestation of stream-of-consciousness reflecting one’s narrative self within time perspective (past-present-future) (Droit-Volet and Dambrun, 2019). Empirical study has also shown that a present hedonism in Zimbardo Time Perspective is associated with less routine in life and the feeling that the previous week passed faster, while a past negative perspective in Zimbardo Time Perspective is related to time pressure, time expansion, and more routine (Wittmann et al., 2015). Importantly, an increased ability of emotional regulation along with a balanced time perspective is associated with experiencing a slower passage of the previous decade (Wittmann et al., 2015).

The third aspect examined by existing research on the feeling of the passage of time (FPT) is the influence of one’s judgment of the speed of time passing on time estimation. Study using ESM (experience sampling method) has shown that the judgment of the speed of time passing affects time estimation down to the level of minutes, that is, the faster that time is perceived as passing, the shorter the estimated time interval, and vice versa – however, this relationship is not maintained at the level of seconds (Droit-Volet et al., 2017). The findings from laboratory experiments also demonstrate that, in the short time range (10–30 s interval), duration estimation and passage of time judgment are distinct temporal processing processes governed by different potential mechanisms (Jording et al., 2022).

The fourth direction regarding the feeling of time passing focuses on psychopathological and phenomenological research on the subjective experience of temporal flow. Studies in this field primarily concentrate on individuals with schizophrenia and depression, employing qualitative research methods such as content analysis (Stanghellini et al., 2016; Vogel et al., 2018, 2019, 2020). The currently accepted consensus suggests that psychotic patients exhibit a disconnection between “past,” “present,” and “future,” resulting in a loss of self-coherence and an inability to generate a continuous stream of consciousness over time (Fuchs, 2013). Therefore, some scholars posit that the generation of temporal stream of consciousness or the emergence of the feeling of time passing may mean the relief of symptoms of schizophrenia (Vogel et al., 2019). In contrast, depressed patients possess distinct concepts of “past,” “present,” and “future” (Stanghellini et al., 2016) and can contemplate their interrelationships at a conscious level. However, at an experience level, depressed individuals often fixate on the past while harboring negativity toward both the present and future. As a result, their perception is characterized by stagnation in relation to the present and future (Thönes and Oberfeld, 2015; Stanghellini et al., 2016), leading to a subjective feeling of slow passage of time (Gallagher, 2012). Nevertheless, since they still retain the concept of “time passing” (the concept of intersubjective temporal experience remains largely intact), this discrepancy between conception and experience further exacerbates their depressive state (Vogel et al., 2018). In summary, there appears to be a fundamental distinction between psychopathic individuals and those suffering from depression concerning how they feel the passage from non-existence to existence (“nothing” versus “there”), even though time seems exceptionally sluggish for patients with depression.

In considering the existing body of research on time passing, there are two points for concern. First, at present, studies mainly examined participants’ judgment of the passage of current time or of short periods of time, but there is lack of investigation into the passage of longer periods of time or of one’s lifetime up to the present. Second, existing studies focus on the perception (i.e., judgment and experience) of the speed of time passing, but lack in-depth investigations of time passing from the perspective of concept or attitude. However, a growing body of research suggests that awareness of the passage of time is more than merely a simple judgment of how fast or slow time is passing (Yu et al., 2017, 2018a,b, 2021). More specifically, first, the feeling of time passing is closely related to self-awareness. Prior research has shown that meditation changes one’s experience of time passing by altering self-awareness, with meditation leading participants to feel that time is passing quickly as they position themselves in a state of “no self” (Droit-Volet and Dambrun, 2019). Second, the feeling of time passing is related to ego identity. Several studies have found that the feeling of time passing is essentially the representation of the state of ego-identity (Yu et al., 2021, 2022). The feeling of time passing also involves elements of social interaction, with several studies suggesting that changes in one’s feeling of the passage of time (FPT) reflect changes in one’s density of experience per standard unit of time (Flaherty et al., 2005; Flaherty, 2018; Yu et al., 2023). Conversely, the empirical density of each standard time unit is constrained by the dynamics of social interactions (Flaherty et al., 2005; Flaherty, 2018). These findings and theories once again show that an individual’s awareness and feeling of the passage of time (FPT) are complex. It is worth noting that some scholars have even proposed more explicitly that the awareness of the passage of time may be a universal way to describe the most significant internal or external environmental changes experienced by an individual (Martinelli and Droit-Volet, 2022). Therefore, it can be speculated that the sense of time passing may be abstract and a comprehensive perception of internal and external environments, psychological feelings, and behavioral reaction tendencies, a concept which this article will hereafter refer to as the View of Time Passing (VTP). The question asked in this study, then, is: what does VTP consist of?

Flavell (1976, 1979) first proposed the concept of metacognition, referring to when cognition, as a cognitive process, becomes the cognitive object itself (Flavell, 1976, 1979; Wang and Guo, 2000). The metacognition of time passing, then, refers to the transition from “feeling of time passing” to “cognition of feeling of time passing.” According to Flavell, metacognition can be understood from two aspects: knowledge about cognition – that is, metacognitive knowledge (MK) – and metacognitive regulation (ME), related to cognitive regulation. The former is a relatively static knowledge system, while the latter is a relatively dynamic activity process. Flavell’s metacognitive concept, especially the concept of MK, provided a theoretical perspective and direction of exploration to begin to answer the question of, “What is the content and structure of view of time passing (VTP)?” Following Flavell’s proposals, this study contends that VTP can also be referred to as the metacognitive knowledge of time passing (MKTP), connoting individual knowledge and intentions about the perceptual process, perceptual results, influencing factors, or other aspects of the feeling of the passage of time.

To our knowledge, there is no existing empirical research on MKTP, especially from the point of view of psychometric verification. Therefore, this study was a first attempt to investigate the content and structure of MKTP from a psychometric perspective. The current study thus aimed to achieve the following: (1) develop a scale to measure MKTP through the use of individual interviews and open-ended surveys, items information collection, and exploratory factor analysis (EFA); (2) analyze the internal *α*, spilt-half, and test–retest reliability of the developed MKTP scale (MKTPS); and (3) analyze the construct-related, convergent, discriminant, criterion-related, and structural validity of the developed MKTPS as assessed through confirmatory factor analysis (CFA).

## Study 1: Scale development

### Methods

#### Interview participants

Sixteen college students (mean age = 19.81, standard deviation = 1.83) from Guangdong Polytechnic Normal University took part in interviews. The sample included eight boys and eight girls, of which three were freshmen, four were sophomores, five were juniors, and four were seniors. The participants were also asked to fill an open-ended survey. All participants signed an informed consent form before beginning the interview.

#### Survey participants

For the aim of exploring the structure of the MKTPS and the selected items, the first developed questionnaire was distributed in two colleges. A total of 996 valid responses were obtained (*M* = 19.41, *SD* = 1.64). In terms of respondent demographics, 264 (26.5%) were male and 732 (73.5%) were female; 546 were freshmen (54.8%), 209 were sophomores (21.0%), 135 were juniors (13.6%), and 106 were seniors (10.6%). The survey was completed online, and participants were informed of the study process before taking part in the study, and were told that their participation was totally voluntary and that their data would be kept confidential. The informed consent form was included in the survey as a document at the beginning of the questionnaire, and the following instructions were presented to the participants: “Please note, before filling the questionnaire, please read the informed consent carefully first. Collection of your data in the survey system means that you have read the informed consent and agreed with it. If you do not wish to participate, you can exit now.”

### Procedure

#### Interviews and open-ended survey

For the goals of exploring the psychological structure of the MKTPS and developing a questionnaire appropriate for college students, three open-ended questions were asked: “Do you feel that time is passing?” “How do you perceive the passage of time?,” and “What does the feeling of the passage of time (FPT) bring to your life?” Please note that these three questions have been formulated based on the first author’s comprehensive analysis of discussions with students regarding the feeling of the passage of time in their daily lives.

Research assistants conducted the interviews and asked the abovementioned questions. The open-ended interview was semi-structured and conducted in a psychological counseling room, with participant answering the questions after having again been provided with detailed, standardized instructions/guidance by the research assistants. The standardized guidance primarily encompasses the following aspects: (1) Participants are encouraged to think independently and respond to the aforementioned three questions without any prompts one by one. (2) In case of a pause during answering a question, it is recommended that participants may choose to skip that particular question and proceed with the next one. If an answer related to the previous question comes to mind while responding to the subsequent question, it can be supplemented at any time. (3) If participants still find it challenging to delve into their thoughts and provide answers after receiving the prompt in (2), they can be further probed regarding the causes, manifestations, and consequences of their feeling of the passage of time. It should be noted that there is no specific pre-set questions in this scenario; instead, questions are creatively formulated and flexibly adjusted based on each individual’s thoughts and responses.

After obtaining informed consent from all participants, the research assistant simply documented the content of interviews in either paper or electronic format. Subsequently, upon completion of all interviews, the research assistants employed Nvivo14 software to systematically organize all interview materials. During the coding process, only responses or excerpts directly or indirectly pertaining to the three questions regarding the feeling of the passage of time, can be incorporated into the corpus. The coding of interview materials in this study was accomplished collaboratively by the two research assistants through frequent communication and exchange, given the abundant use of rhetoric and metaphor in the interview materials.

#### Data collection

After analyzing the interview results, 121 valid descriptions of MKTP were obtained. Two research assistants worked together to analyze the descriptions and generated four categories for the 121 descriptions. The first category related to the promotion of positive emotion (8.26% of items). The second category represented the inhibition of negative emotion (9.09% of items). The third category reflected the relationship between life scenarios and the formation of the feeling of time passing (47.11% of items). The fourth category referenced the effects experienced after activating the feeling of time passing (35.54% of items). The representative statements of each category are presented in Table 1. The structure of the MKTPS was therefore hypothesized to include four factors of the feeling of time passing: positive promotion (PP), negative inhibition (NI), life touching (LT), and priming effects (PE).

**Table 1:** Open coding and categorization of college students’ view of time passing.

Category	Source material (partial)	Reference points	Percent (%)
Positive promotion	If I am engaged in activities that pique my interest, such as immersing myself in a book or engaging in sports, I perceive the passage of time to be accelerated.	10	8.26
	The night came to an end as I enjoyed a lively gathering with my companions.		
	When you are enjoying a good meal or going out to play, you will feel that the time will pass quickly.		
Negative inhibition	When you are in a bad mood, you feel that time passes slowly, and when you are bored, you feel that time passes slowly.	11	9.09
	When stretching (painfully), the last 30 s always feel like a minute.		
	If I am in a sad or painful state, I will feel that time passes slowly.		
Life touching	When I look back, if I feel like I spent each time meaningfully, then I achieved something.	57	47.11
	When I think about the passage of time, I think about the time until death, what I have already accomplished, and what I can do with the remaining time.		
	When your life is very full, your sense of the passage of time may be weak. When you have nothing to do every day, the sense of time passing is very strong.		
Priming effects	I’m more productive when I feel time passing.	43	35.54
	The feeling of time passing affects my living arrangements.		
	The feeling of time passing makes me think about a lot of things, life, meaning, things like that.		

#### Development of the preliminary MKTPS

Using the above-mentioned four-factor structure, 40 items were created at one time. Seven undergraduates evaluated, discussed and revised the items. Then, two associated professors and one graduate student in department of applied psychology further assessed the items according to the dimensions that each item was intended to evaluate. As a result, 35 items were developed into a preliminary MKTPS, each of which was rated using a five-point Likert scale ranging from 1 (completely disagree) to 5 (completely agree).

Because the current study sample involved participants sourced from several universities and an online measurement, a protocol was followed to ensure that all data was collected in a similar fashion. First, direct contact was made with each university, and the experimenter was given an opportunity to clarify any queries regarding the purpose, objectives, timing, or content of this research. A brief online meeting was held with all participating course teachers from the different universities, and a convenient testing schedule was finalized. With regards to ethics, the present study was approved by the ethics committee of school of education science of Guangdong Polytechnic Normal University. All participants were treated in accordance with the American Psychological Association’s code of ethics, which includes confirming participants’ informed consent, a confidentiality agreement, and a statement of anonymity. All participants were informed of the process at beginning of the study, and it was underscored that their participation was completely voluntary and that their data would be kept confidential. The informed consent form was included as a document at the beginning of the survey (for the specific insertion of the informed consent form, see “*Survey Participants*” above).

### Results

#### Item analysis

All the items of the preliminary MKTPS showed good item-discrimination indices, demonstrating that each high scoring item (bottom 27%) on the scale differed significantly (*p* < 0.001) from each low-scoring item (top 27%). The majority of corrected item-total correlations were greater than 0.40, with only four of the items lower than 0.40. After deleting these four items, the item reliability indices ranged from 0.438 to 0.685, offering evidence that the items exhibited acceptable internal consistency and generated a good distribution of responses.

#### Exploratory factor analysis (EFA)

Exploratory factor analysis was conducted using principal components analysis (PCA) with varimax rotation to explore the dimensionality of the MKTPS. The appropriateness of the factor model was assessed based on the Kaiser–Meyer–Olkin index (KMO = 0.94) and Bartlett’s Test of Sphericity (11060.67, *df* = 231, *p* < 0.001), providing that the items shared common factors. Four steps were taken to decide the number of factors to stand stable. First, the scree plot was drawn and tested, and a model with the same number of common factors as the number of eigenvalues prior to the last substantial drop was then fit to the data. Second, the eigenvalues of the factors were calculated, and needed to be greater than 1.00. Third, the variance explained was assessed, which needed to be greater than 3% for each extracted factor before rotation. Fourth, the factor was checked, and needed to contain at least three items. Invalid items were deleted if they did not satisfy two criteria: that communalities were <0.30, and that the highest factor loading of an item in absolute value on one factor was <0.45. Finally, four distinct factors were obtained, with 62.86% of the total variance explained and 22 items loading highest on each retained factor (loading values varying from 0.57 to 0.84; see Table 2). The EFA showed the following four distinct MKTPS factors (i.e., scale items; see Table 2).

**Table 2:** Items retained after exploratory factor analysis and the corresponding load indicators.

Number	Item	Factor 1	Factor 2	Factor 3	Factor 4	Communality
18	FTP affects the pace of my life.	**0.824**	0.156	0.093	0.151	0.735
20	FTP affects my time decisions.	**0.790**	0.186	0.112	0.127	0.688
19	FTP affects my estimation of the length of time in the future.	**0.782**	0.222	0.103	0.142	0.691
22	FTP affects the way I think.	**0.759**	0.161	0.094	0.081	0.617
23	FTP affects my judgement of the length of my lifetime.	**0.724**	0.280	0.117	0.122	0.631
21	FTP affects my estimation of the length of time in the past	**0.710**	0.249	0.154	0.126	0.605
24	FTP affects my financial decisions.	**0.680**	0.226	0.004	0.114	0.527
17	FTP affects my estimation of how long time is in the present moment.	**0.672**	0.202	0.223	0.205	0.584
12	FTP affects my plan of action/s.	**0.573**	0.151	0.091	0.245	0.420
28	I have a strong sense of FTP as I realize that I still cannot achieve what I want in my life.	0.276	**0.757**	0.101	0.139	0.678
25	When I am in a frustrated state, I will lament the passage of time.	0.234	**0.705**	0.168	0.169	0.608
27	Recalling the past always gives me a strong sense of FTP.	0.208	**0.698**	0.275	0.007	0.606
29	When I realized that life will eventually end, I had a strong sense of FTP.	0.281	**0.670**	0.184	0.100	0.572
26	I often lament the passage of time for minor reasons.	0.307	**0.594**	−0.041	0.178	0.481
3	When I am excited, I feel like that time is passing quickly.	0.105	0.128	**0.843**	0.113	0.752
1	When I am happy, I feel like that time is passing quickly.	0.106	0.126	**0.805**	0.095	0.685
7	When I am joyful, I feel like that time is passing quickly.	0.135	0.203	**0.776**	0.175	0.692
5	When I am delighted, I feel like that time is passing quickly.	0.153	0.082	**0.680**	0.234	0.547
13	When I am sad, I feel like that time is passing very slowly.	0.228	0.094	0.108	**0.832**	0.764
11	When I am frustrated, I feel like that time is passing slowly.	0.164	0.188	0.140	**0.797**	0.718
9	When I am depressed, I feel like that time is passing slowly.	0.109	0.112	0.170	**0.755**	0.623
15	When I am in pain, I feel like that time is passing slowly.	0.271	0.101	0.223	**0.688**	0.607
Eigenvalues		5.34	2.89	2.82	2.78	
Explained variance (62.86%)		24.26	13.15	12.81	12.63	

FTP = the feeling of the passing of time; bold/underlined formatting indicates the loadings after converging to a factor through rotation. The order of the items is based on the EFA running result, not the original order.

##### Factor 1: Priming effect (PE)

The nine items from the first factor measure the respondent’s understanding of what happens to their life as they experience the passage of time. Through careful identification of these nine items, we found that, in the participants’ mind, the feeling of time passing has an impact on all aspects of daily life, affecting their way of thinking, their estimation of the length of their daily life, and the organization and planning of daily life.

##### Factor 2: Positive promotion (PP)

Four items assessed the respondent’s awareness of positive emotions causing respondents to feel like time is passing quickly.

##### Factor 3: Negative inhibition (NI)

These four items reflect the respondent’s awareness that negative emotions make people feel like that time is passing slowly.

##### Factor 4: Life touching (LT)

This factor contains five items that express the correlation between the feeling of time passing and the respondent’s state, meaning, and quality of life.

## Study 2: Reliability and validity analysis of the MKTPS

### Participants

Participants were recruited from four colleges in Guangdong and Shaanxi Province to complete the newly-developed MKTPS. 1, 671 valid responses (across all four samples) was obtained. The data of these four samples were used to perform confirmatory factor analysis, internal consistency reliability analysis, measurement invariance testing, and assess construct-related validity, retest reliability, and criterion association validity. Table 3 shows the specific details of each sample group, and their data usage.

**Table 3:** Information regarding the total Sample size of Study 2.

Sample number	Data used for	Total number	Sex		Grade				Age range (*M* ± *SD*)
			Male	Female	Freshman	Sophomore	Junior	Senior	
Sample 1	Confirmatory factor analysis, internal consistency reliability analysis, split-half reliability analysis, measurement invariance, construct-related validity	770	248 (32.2%)	522 (67.8%)	352 (45.7%)	126 (16.4%)	224 (29.1%)	68 (8.8%)	19.87 ± 1.79
Sample 2	Retest reliability, internal consistency reliability analysis	84	20 (23.8%)	64 (76.2%)	14 (16.7%)	34 (40.5%)	24 (28.6%)	12 (14.3%)	20.26 ± 1.53
Sample 3	Criterion validity 1, internal consistency reliability analysis	454	111 (24.4%)	343 (75.6%)	263 (57.9%)	123 (27.1%)	36 (7.9%)	32 (7.0%)	19.09 ± 1.33
Sample 4	Criterion validity 2, internal consistency reliability analysis	363	106 (29.2%)	257 (70.8%)	152 (41.9%)	105 (28.9%)	51 (14.0%)	55 (15.2%)	19.77 ± 1.23

### Measures

The following measures were applied to test the validity of the MKTPS.

#### Zimbardo time perspective inventory (ZTPI)

The ZTPI was developed by Zimbardo and Boyd (1999) and contains 56 questions which measure five dimensions of time perspective: Past–Negative (10 questions), Present–Hedonistic (15 questions), Future (13 questions), Past–Positive (9 questions), and Present–Fatalistic (9 questions). The Chinese version of the ZTPI questionnaire (Tao et al., 2015) was utilized in the present study. Examples of items are “I believe that getting together with one’s friends to party is one of life’s important pleasures” and “Familiar childhood sights, sounds, and smells often bring back a flood of wonderful memories.” Each question is evaluated using a five-point Likert scale, ranging from 1 (very uncharacteristic of me) to 5 (very characteristic of me). Items 9, 24, 25, 41, and 56 are reverse-scored. The Cronbach’s αs for the five dimensions in the current study were 0.818, 0.726, 0.655, 0.709, and 0.742, respectively.

#### Time management disposition inventory (TMDI)

Time management disposition was assessed using the TMDI, which was developed by Huang and Zhang (2001). It contains 44 items, each of which is assessed using a five-point Likert scale ranging from 1 (totally disagree) to 5 (totally agree) and evaluates three dimensions of time management disposition: sense of time value (STV), sense of time control (STC), and sense of time efficacy (STE). Example items are “Timing is everything for everyone” and “I believe that time is life.” The Cronbach’s αs for the three dimensions in the present study were 0.794, 0.881, and 0.814, respectively.

### Procedure

The questionnaires used in Study 2 were completed online, following the same ethical review, informed consent, and research procedures as used in Study 1.

### Results

#### Reliability

Cronbach’s *α* and test–retest reliability were employed to evaluate the reliability of the MKTPS and its dimensions. The Cronbach’s αs were calculated for the data collected from all four samples used in Study 2, and detailed results are presented in Table 4. Using the data collected from Sample 1, the half-reliability of the total scale and all four dimensions were 0.75, 0.90, 0.80, 0.81, and 0.83, respectively. The data collected from Sample 2 was used to assess the retest reliability, and the test–retest reliability of the whole scale and all four factors were 0.823, 0.795, 0.645, 0.793, and 0.761, respectively. All the reliability coefficients were greater than 0.60, indicating that the MKTPS possesses acceptable internal consistency and stability over time.

**Table 4:** Cronbach’s αs of the MKTPS and its subscales.

Sample	Priming effect	Positive promotion	Negative inhibition	Life touching	Total scale
Sample 1	0.90	0.84	0.84	0.80	0.91
Sample 2	0.83	0.80	0.90	0.64	0.83
Sample 3	0.89	0.81	0.84	0.74	0.89
Sample 4	0.92	0.87	0.89	0.85	0.95

MKTPS, metacognitive knowledge of time passing scale.

### Validity

#### Construct-related validity

Measurement theory holds that construct validity is confirmed when the correlations between the factors and the whole scale are greater than the correlations between each dimension. As revealed in Table 5, the absolute values of the correlations between each dimension and the whole scale fell between 0.64 and 0.87, and were greater than the correlations between each dimension, suggesting that the MKTPS has good construct validity.

**Table 5:** Correlations between dimensions and whole scale (sample 1 of Study 2, *n* = 770).

Variable	*M*	*SD*	1	2	3	4	5
1. Priming effect	30.67	5.51	1				
2. Positive promotion	15.47	2.67	0.33^**^	1			
3. Negative inhibition	13.55	2.75	0.41^**^	0.40^**^	1		
4. Life touching	17.55	3.27	0.60^**^	0.41^**^	0.39^**^	1	
5. Total scale	77.24	10.92	0.87^**^	0.64^**^	0.67^**^	0.80^**^	1

M, mean; SD, standard deviation. ^**^p < 0.01.

#### Convergent validity

Convergent validity was evaluated using the correlations between the MKTPS subscales and the past perspective subscale of the ZTPI. As shown in Table 6, each MKTPS subscale was significantly positively correlated with each subscale of the ZTPI. Considering that MKTP is a view of the past time passing, it should correlate highly with the past time view scale of the ZTPI (Zimbardo Time Perspective Inventory). In fact, with the exception of the Positive Promotion (PP) subscale, the correlation coefficients between the rest MKTPS subscales (i.e., PE, NI, and LT) and the Negative Past ZTPI subscale were relatively high, and more specifically, higher than the correlation coefficients between these MKTPS subscales (i.e., PE, NI, and LT) and the other Zimbardo subscales (i.e., present–hedonistic, present fatalistic, and future).

**Table 6:** Results of convergent and discriminant validity analyses of the MKTPS (Sample 3 in Study 2, *n* = 454).

	Pa–Ne	Pr–He	Fu	Pa–Po	Pr–Fa	PE	LT	PP	NI	MKTP
Pa–Ne	1									
Pr–He	0.32^**^	1								
Fu	−0.12^*^	0.11^*^	1							
Pa–Po	−0.18^**^	0.27^**^	0.29^**^	1						
Pr–Fa	0.53^**^	0.33^**^	−0.20^**^	−0.17^**^	1					
PE	0.31^**^	0.25^**^	0.14^*^	0.02	0.18^**^	1				
LT	0.41^**^	0.36^**^	0.14^**^	0.15^**^	0.21^**^	0.56^**^	1			
PP	0.13^**^	0.25^**^	0.07	0.22^**^	0.08	0.28^**^	0.28^**^	1		
NI	0.17^**^	0.10	0.15^**^	0.14^**^	0.12^**^	0.29^**^	0.30^**^	0.39^**^	1	
MKTP	0.37^**^	0.33^**^	0.18^**^	0.15^**^	0.22^**^	0.86^**^	0.76^**^	0.57^**^	0.62^**^	1

Pa–Ne, past–negative; Pr–He, present–hedonistic; Fu, future; Pa–Po, past–positive; Pr–Fa, present–fatalistic; PE, priming effect; LT, life touching; PP, positive promotion; NI, negative inhibition; MKTP, metacognitive knowledge of time passing. ^*^p < 0.05, ^**^p < 0.01.

#### Discriminant validity

Discriminant validity was rated using the correlations between the MKTP subscales and the future perspective of the ZTPI. As shown in Table 6, each dimension of the MKTPS was either weakly or not at all related with the future perspective of the ZTPI, providing evidence for discriminant validity.

#### Criterion-related validity

Previous research has suggested that there is a significant positive correlation between college students’ emotions regarding and attention to the passage of time and their time management deposition (Yu et al., 2021, 2022). In the current study, the total scale as well as all four MKTPS subscales were strongly associated with the total and subscale scores of the TMDI, providing evidence for good criterion validity of the MKTPS. For details, see Table 7.

**Table 7:** Results of criterion-related validity analysis of the MKTPS (Sample 4 in Study 2, *n* = 363).

	1	2	3	4	5	6	7	8	9
1. PE	1								
2. LT	0.70^**^	1							
3. PP	0.51^**^	0.54^**^	1						
4. NI	0.62^**^	0.59^**^	0.53^**^	1					
5. MKTP	0.91^**^	0.85^**^	0.73^**^	0.80^**^	1				
6. STV	0.49^**^	0.46^**^	0.36^**^	0.38^**^	0.51^**^	1			
7. STC	0.35^**^	0.28^**^	0.30^**^	0.26^**^	0.37^**^	0.66^**^	1		
8. STE	0.32^**^	0.31^**^	0.33^**^	0.27^**^	0.37^**^	0.62^**^	0.80^**^	1	
9. TMD	0.42^**^	0.36^**^	0.36^**^	0.31^**^	0.45^**^	0.81^**^	0.96^**^	0.89^**^	1

PE, priming effect; LT, life touching; PP, positive promotion; NI, negative inhibition; MKTP, metacognitive knowledge of time passing; STV, sense of time value; STC, sense of time control; STE, sense of time efficacy; TMD, time management deposition. ^**^p < 0.01.

#### Confirmatory factor analysis (CFA)

**Figure 1:**
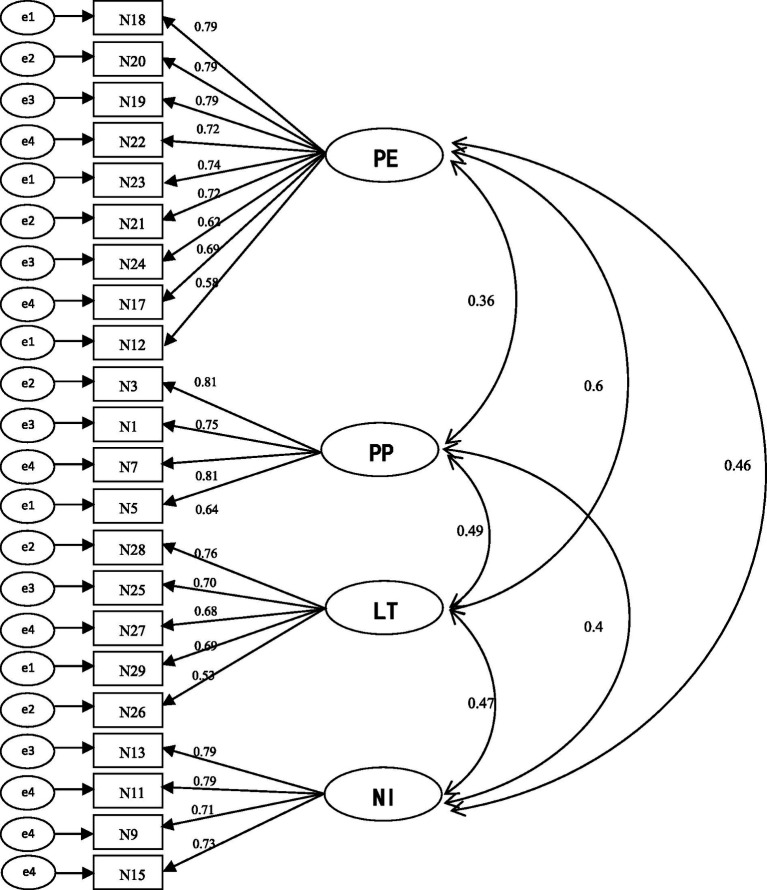
Structure diagram of the four factors of the MKTPS. PE = view of priming effect, PP = view of positive promotion, LT = view of life touching, NI = view of negative inhibition.

Confirmatory factor analysis was executed using Mplus 8.30 to assess the goodness-of-fit of the model to the data. Based on the EFA results, we expected the model to contain four dimensions. Each dimension was measured by the items specific to it, with the errors of each dimension not relating with one another. Overall, the four-factor structure of the MKTPS showed a good fit result, satisfying the psychometric indices (see Table 8 for fitting indices of the structural equation). Each item had a high load on its corresponding factor (the standardized load of Sample 3 was between 0.531 and 0.809), and all items were found to be valid (see Figure 1 for the verification structure of Sample 3 data). We then tried to reduce the number of factors to construct a more concise model. These three reduced models are presented in Table 8: *Three factors (1)* refers to the combination of PP and NI, while PE and LT remain unchanged; *Three factors (2)* refers to the combination of NI and LT, while PE and PP remain unchanged; *Three factors (3)* refers to the combination of PP and LT, while PE and NI remain unchanged. *The two factors model* refers to the combination of PP, NI, and LT, while PE remains unchanged. All models with reduced factors were compared to the four-factor model. As Table 8 shows, the fitting results of the four-factor model were relatively better than the other five models.

**Table 8:** Comparison of comparative factor analysis results of Sample 1 of Study 2 (*n* = 770).

Model	*χ* ^2^	df	*χ*^2^/*df*	RMSEA	CFI	TLI	SRMR
**Four-factor**	**399.452** ^***^	**203**	**1.97**	**0.035**	**0.961**	**0.956**	**0.040**
Three-factor (1)	1013.020^***^	206	4.92	0.071	0.840	0.821	0.061
Three-factor (2)	928.012^***^	206	4.50	0.067	0.857	0.840	0.065
Three-factor (3)	931.311^***^	206	4.52	0.068	0.856	0.839	0.069
Two-factor	1365.493^***^	208	6.56	0.085	0.771	0.746	0.084
One factor	1942.368^***^	209	9.29	0.104	0.657	0.621	0.103

The bold font denotes the optimal model and its corresponding fit index. RMSEA, root-mean-square error of approximation; CFA, comparative fit index; TLI, Tucker–Lewis index; SRMR, standardized root mean square residual. ^***^p < 0.001.

#### Measurement invariance (MI)

A recent study of Yu et al. (2022) revealed that there are significant differences in the feeling of time passing in terms of genders, grades, and majors. Therefore, using the data of Sample 3, we tested the measurement invariance (MI) of the MKTPS with regards to gender, grade, and major. Four kinds of tests were conducted – configural, metric, scalar, and strict measurement invariance – and the results showed no significant differences between different genders, grades, and majors. As Table 9 shows, the results of the data analyses supported the configural invariance of the five-dimensional structure of the MKTPS in terms of gender, grade, and major, that is, all models fit well. Meanwhile, metric, scalar, and strict metric invariance were also supported, and the variations of CFI and RMSEA did not exceed the proposed critical values of ΔCFI ≤0.01 and ΔRMSEA ≤0.015 (Cheung and Rensvold, 2002).

**Table 9:** Measurement invariance analysis results (Sample 1 of Study 2, *n* = 770).

Model	*χ* ^2^	df	CFI	RMSEA	MC	ΔCFA	ΔRMSEA
Gender							
M0 (configural)	601.246	406	0.962	0.035			
M1 (metric)	630.264	424	0.960	0.036	M_0_ vs. M_1_	0.002	−0.001
M2 (scalar)	660.121	442	0.958	0.036	M_1_ vs. M_2_	0.002	0.000
M3 (strict)	678.055	464	0.958	0.035	M_2_ vs. M_3_	0.000	0.001
Grade							
M0 (configural)	1326.956	812	0.917	0.058			
M1 (metric)	1362.717	866	0.920	0.055	M_0_ vs. M_1_	0.003	−0.003
M2 (scalar)	1419.462	920	0.919	0.053	M_1_ vs. M_2_	−0.001	−0.002
M3 (strict)	1547.057	986	0.909	0.055	M_2_ vs. M_3_	−0.01	−0.02
Major							
M0 (configural)	662.456	406	0.954	0.041			
M1 (metric)	689.403	424	0.952	0.040	M_0_ vs. M_1_	−0.002	−0.001
M2 (scalar)	707.888	442	0.952	0.040	M_1_ vs. M_2_	0.000	0.000
M3 (strict)	751.118	464	0.948	0.040	M_2_ vs. M_3_	−0.004	0.000
Residence							
M0 (configural)	1169.995	812	0.939	0.048			
M1 (metric)	1229.961	866	0.938	0.047	M_0_ vs. M_1_	−0.001	−0.001
M2 (scalar)	1283.061	920	0.938	0.045	M_1_ vs. M_2_	0.000	−0.002
M3 (strict)	1329.664	986	0.941	0.043	M_2_ vs. M_3_	0.003	−0.002

CFI, comparative fit index; SRMR, standardized root-mean-square residual; RMSEA, root-mean-square error of approximation; MC, model comparison; ΔCFI, M_n-1_’s CFI-M_n_’s CFI; ΔRMSEA, M_n-1_’s RMSEA-M_n_’s RMSEA.

## Discussion

### The psychological structure of the MKTP

The feeling of the passage of time (FTP) is a new horizon of time psychology, with current research still only in the initial stages (Yu et al., 2018a). Empirical studies on one’s view of time passing (or one’s awareness of time passing from a metacognitive perspective) is relatively less. However, from the perspective of daily life, the view of time passing or the MKTP will affect one’s time estimations and decision-making, thus affecting their long-term time or life management. Therefore, it is necessary to understand the content and structure of the view of one’s lifetime passing. Meanwhile, current research methods and equipment available in the investigation of the psychological processing of time is only able to place a focus on the field of consciousness or view, leading us to carry out the present study as an attempt to develop a psychometric tool to explore it.

As mentioned previously, Western scholars are more likely to define the feeling of time passing as a cognitive judgment of time passing (Droit-Volet and Wearden, 2016; Droit-Volet et al., 2017), however this does not fully align with Chinese interpretations of the passage of time. As Lv Zhengmeng, a Song Dynasty scholar in ancient China, put it: “Time is the combination of your life experiences, your destiny, and your fortune.” According to the theoretical analysis of Jhou (2020) and Su and Ye (2020), Chinese people do not view time from a single perspective, but rather embrace it as encompassing space, physicality, and interpersonal relationships. Obviously, the Chinese people’s feeling of the passage of time is undoubtedly profound and multifaceted. This complex and profound experience of the passage of time was reflected in the interview responses gathered in the current study, for example, “I feel that many external factors affect my feeling of time passing, but I also want to know how I evaluate it in my heart under the influence of the outside world” or “I think it (FPT) is a kind of scale that can be used to measure life and growth.”

Based on the interviews performed, the MKTPS and its four subscales were developed: view of life touching (LT), priming effect (PE), positive promotion (PP), and negative inhibition (NI). LT refers to one’s perception of the factors influencing their feeling of the passage of time, PE is one’s cognition of the aftereffects of the feeling of the passage of time, while PP and NI encompass one’s cognition of the changes involved and feelings processed in experiencing the passage of time, respectively. Together, these four subscales essentially cover Flavell’s complete depiction of metacognitive knowledge (MK). The LT and PE are likely to be more pronounced in Chinese adults other than college students. This is supported by a review study indicating that as individuals’ age, their concept of time becomes broader, more variable, and characterized by greater sense of self-continuity (Li-Jun et al., 2023). These aspects can be reflected in the LT and PE examined in the present study. Furthermore, the PP and NI investigated here align with findings from Western studies on metacognition of feeling of the passage of time (i.e., the emotion-attention model of metacognition of time passing) (Lamotte et al., 2014). The cultural applicability and generalizability of these results will be discussed further along with specific research conclusions.

In his seminal work Being and Time (Sein und Zeit), Heidegger put forward that human beings are more aware of their finiteness and limitations then they are of death (Heidegger, 1927; Droit-Volet and Dambrun, 2019). Such awareness inevitably affects one’s judgment of the passage of time (Neuringer and Harris, 2011; Droit-Volet and Dambrun, 2019). Interestingly, this viewpoint is reflected in the dimension of view of life touching (LT) in the present study. For instance, one respondent told us, “When I realize that my life will ultimately end, I feel a strong sense of time passing.” Moreover, Kosak et al. (2019) also argued that the perception of time passing is based on memory activation, and this activation must have a certain heuristic significance. This is similarly reflected in the items measuring the LT dimension in the present study, such as “Recalling the past always makes me feel a strong feeling of time passing.” Overall, the findings of the current study as well as existing literature indicate that, at a conceptual level, individuals seem to attribute the feeling of the passage of time to higher concepts such as life consciousness and life meaning – an understanding also supported by philosophers who have suggested that differences or changes in one’s feeling of the passage of time is rooted in fluctuations of meaning (August, 2018).

Regarding college students’ understanding of the PE dimension of the MKTPS, that is, one’s view of aftereffects on the feeling of passage of time, the current study findings are consistent to existing empirical results. Droit-Volet et al. (2017, 2018) tested the relationship between the judgment of the passage of time and time estimation at a minute level, i.e., ranging from 2 to 8 min in one experiment, and from eight to 32 min in another and found that the feeling of time passing is indeed related to time estimation in that the faster the feeling of time passing, the shorter one’s time estimation. The feeling of time passing is a good predictor of the “verbal estimation of long time distance.” This correlation suggests that there is a common mechanism between the two ways of perceiving time at this scale. The judgment of the passage of one’s lifetime is necessarily related to one’s long-term memory, and the retrospective judgment of time is, to a large extent, the reconstruction of non-temporal information in one’s memory, such as numerous perceptual changes, memory load, and activity segmentation. These retrospective memories may also play a key role in our awareness or view of the passage of time (Droit-Volet and Dambrun, 2019).

Numerous studies have shown that one’s current emotional or arousal state affects their feeling of the passage of time (Martinelli and Droit-Volet, 2022, 2023; Liu et al., 2023), with findings showing that participants who report feeling happy report time passing quickly (Droit-Volet et al., 2021), while participants who report feeling frustrated report time passing slowly (Tipples, 2018). It is noteworthy that in a qualitative study on the “experience of time,” when discussing the feeling of time passing, most participants explicitly acknowledged the influence of situational and social contexts on their temporal experiences. These situations and social contexts often encompass interesting and/or pleasant or stressful activities or situations, wherein time tends to elapse more swiftly, particularly in interpersonal settings (Vogel et al., 2020). However, few empirical studies have examined the attribution and cognition of this phenomenon from the perspective of metacognition. In the present study, most participants were able to recognize the effects of emotion and arousal on their feeling of the passage of time. In terms of the specific connotations, the PP (positive promotion) dimension reflects primitive emotions with high arousal, while NI (negative inhibition) reflects secondary self-conscious emotions with low arousal. This inclusion of these dimensions in the MKTPS is partially consistent with the inclusion of emotion and attention in the metacognitive theory of time passing (Lamotte et al., 2014), which posits that “positive states promote the feeling of time passing quickly, and negative states induce the feeling of time passing slowly.” Different to Lamotte et al. (2014), however, the current study integrated emotion and arousal items into one dimension, while Lamotte et al. (2014) measured the dimensions of emotion and attention separately. In the context of Chinese college students specifically, while they can consider the impact of both positive and negative states on the feeling of time passing at a conceptual level, the impact of negative states is often more prominent at the levels of perception and experience (Yu et al., 2017, 2018b, 2021).

### The reliability and validity of the MKTPS

The internal consistency reliability for the four subscales of MKTPS as developed in the current study was uniformly good. The majority of the calculated Cronbach αs were above 0.60 (see Table 4), and the internal consistency of the scale as a whole ranged from 0.83 to 0.91 for Samples 1 to 4, confirming the good psychometric properties of the MKTPS. The test–retest reliability of the MKTPS was examined using an independent sample of 84 students with a four-week interval. The autocorrelations ranged from 0.65 to 0.80, and the test–retest reliability of the whole scale was 0.82. Thus, the temporal reliability of the scales was established.

The results of CFA supported our model of the MKTPS as represented by four latent factors. The items of the scale had high latent factor loadings where expected (Qiu and Lin, 2019), and all four factors loaded highly onto the MKTP (0.53–0.81). The goodness-of-fit index, *χ*^2^/*df* = 1.97, was lower than the threshold value (2.0), as proposed by Qiu and Lin (2019). With the exception of the four-factor model, all models tested in the current study showed model fit index problems, for example, *χ*^2^/*df* was greater than 2 (all models excluding the four-factor model), the RMSEA was greater than 0.08 (two-factor and single-factor models), the CFI and TLI were lower than 0.90 (all models excluding the four-factor model), or the SRMR was higher than 0.08 (two-factor and single-factor models). As such, the four-factor model was determined to be the best model with the best fit.

Regarding construct validity, the correlations between the factor and the whole scale scores were evaluated (as illustrated in Table 5), and the absolute values of the correlations between each factor and the whole scale were between 0.64 and 0.87, and greater than that of the correlations between each dimension, demonstrating good initial construct validity. Convergent validity and discriminant validity were also taken into account. There existed a significant positive correlation between the past view of the ZMPI and the MKTPS, and the correlation coefficient between the ZMPI negative past and the MKTPS was greater than that between the positive past and the MKTPS. This further indicates that Chinese college students generally have a more negative attitude toward the past and the passage of time, which may also be related to the tendency of Chinese people tendency to form negative judgments about their self at past (Li et al., 2021; Zhang et al., 2021). Discriminant validity was also proved by our results, with low but significant correlations found between the ZMPI future perspective and the MKTPS total and subscales (see Table 6). This pattern of correlations suggests that the total MKTPS and its subscales each measure different constructs.

Regarding criterion validity, the total MKTPS and its four subscales correlated significantly with the total TMDI and its three subscales, providing good evidence of criterion validity. First, the total score of the MKTPS in this study was highly significantly positively correlated with that of the TMDI (*r* = 0.45). This shows the close connection between cognition and behavior and the feeling of the passage of time, which is in line with the philosophical hypothesis put forward by Young (2022) regarding “the relationship between embodied action and the concept/attitude of time”. In fact, a significant positive correlation was found not only between the MKTPS and the TMDI, but also between the experience of time passing (ETP) and the TMDI (Yu et al., 2021, 2022). It is worth noting that of the four dimensions of the MKTPS, PE, which reflects one’s behavioral tendencies of outcome-oriented time regulation, has the highest correlation with the TMDI (*r* = 0.42). Meanwhile, the correlation coefficients between STV (sense of time value) and the four dimensions of the MKTPS were higher than those between the other two dimensions of the TMDI (i.e., time control and time efficacy) and the four dimensions of the MKTPS. Considering that STV relates to the conceptual level (Huang and Zhang, 2001), this finding further indicates that the content of the MKTPS is also subordinate to the conceptual level.

### Research limitations and future directions

People of different ages have different views about the passage of time according to the unique experiences. The current study focused specifically on college students as its participants, and the distributions of gender, grade, and other demographic variables were relatively unbalanced, even though the measurement invariance of the MKTPS in these demographic variables was valid. In the future, however, the structure of the view of time passing should be explored in other age groups, with consideration of balancing the distributions of demographic variables such as gender, ethnicity, and family socioeconomic status. As the conclusions drawn in the present study are based only on Chinese college students, cross-cultural research should be carried out to enrich our understanding of the complexities of the view of time passing. In the specific context of Western culture, it is imperative to investigate whether the “conception of time passing” among Western individuals also encompasses an awareness of the “consequences resulting from the feeling of time passing,” as well as “whether this feeling can or to what extent to influence the contemplation on life’s significance.” In addition, a recent study examined the conceptual structure of “metacognitive experience of time passing” from the perspective of “metacognitive experience” and developed corresponding assessment tool (Yu et al., 2023). It is necessary to conduct future testing on the newly developed “Metacognitive Knowledge of Time passing Questionnaire” in the present study and “Metacognitive Experience of Time Passing Questionnaire,” in order to investigate the specific relationship between metacognitive experience and metacognitive knowledge of time passing.

Assessing the perception of the passing of time can be done in many ways in addition to the dimension of “fast vs. slow,” such as “presence vs. absence,” “deep vs. shallow,” and “far vs. near.” The items in the present study mainly examined “fast vs. slow,” however, which ultimately is insufficient. Culturally, Western society places more emphasis on linear time culture, while Eastern cultures are more likely to embrace cyclical time culture. How, then, does the view of the passage of time differ between these two different cultures? What are the structural similarities and differences? Research has shown that individuals with a linear view of time are more likely to prefer short-term options when making intertemporal decisions compared with those with a cyclical view of time (Xu et al., 2019), highlighting one example of the value of developing comprehensive tools to measure how individuals experience and perceive the passing of time. As such, future optimization of the MKTPS could consider the addition of related dimensions.

## Conclusion

The four-factor structure of the MKTP scale has been verified, comprising positive emotion priming (PP), negative arousal inhibition (NI), priming effect (PE), and life touching (LT). The scale is shown to possess good reliability and validity among Chinese college students, meeting the psychometric requirements and can be considered for use as an assessment tool of the basic attitudes and views of time passing in Chinese college students.

## Data Availability

The raw data supporting the conclusions of this article will be made available by the authors, without undue reservation.
